# Pathological tumor infiltrative pattern and sites of initial recurrence in stage II/III gastric cancer: Propensity score matching analysis of a multi‐institutional dataset

**DOI:** 10.1002/cam4.1868

**Published:** 2018-11-08

**Authors:** Nobuhiko Nakagawa, Mitsuro Kanda, Seiji Ito, Yoshinari Mochizuki, Hitoshi Teramoto, Kiyoshi Ishigure, Toshifumi Murai, Takahiro Asada, Akiharu Ishiyama, Hidenobu Matsushita, Chie Tanaka, Daisuke Kobayashi, Michitaka Fujiwara, Kenta Murotani, Yasuhiro Kodera

**Affiliations:** ^1^ Department of Gastroenterological Surgery (Surgery II) Nagoya University Graduate School of Medicine Nagoya Japan; ^2^ Department of Gastroenterological Surgery Aichi Cancer Center Nagoya Japan; ^3^ Department of Surgery Komaki Municipal Hospital Komaki Japan; ^4^ Department of Surgery Yokkaichi Municipal Hospital Yokkaichi Japan; ^5^ Department of Surgery Konan Kosei Hospital Konan Japan; ^6^ Department of Surgery Ichinomiya Municipal Hospital Ichinomiya Japan; ^7^ Department of Surgery Gifu Prefectural Tajimi Hospital Tajimi Japan; ^8^ Department of Surgery Okazaki City Hospital Okazaki Japan; ^9^ Department of Surgery Tosei General Hospital Seto Japan; ^10^ Biostatistics Center, Graduate School of Medicine Kurume University Kurume Japan

**Keywords:** gastrectomy, gastric cancer, pathological tumor infiltrative pattern, recurrence

## Abstract

**Background:**

Advanced gastric cancer frequently recurs even after radical resection followed by adjuvant chemotherapy. The aim of this study was to evaluate the relationship between pathological infiltrative pattern (INF) and initial recurrence patterns in patients with stage II/III gastric cancer using a large multicenter database.

**Methods:**

We retrospectively analyzed 1098 eligible patients who underwent curative gastrectomy for stage II/III gastric cancer at nine institutions between 2010 and 2014. Patients were categorized into the INF‐a/b and INF‐c groups and adjusted using propensity score matching.

**Results:**

After propensity score matching, 686 patients (343 for each) were classified in the INF‐a/b and INF‐c groups. There were no significant differences in overall and disease‐free survival between the two groups. In the INF‐a/b group, frequencies of recurrence at the peritoneum, lymph node, and liver were equivalent. In contrast, the peritoneum was the most frequent site and accounted for 60% of the total recurrences in the INF‐c group. The cumulative peritoneal recurrence rate was significantly higher in the INF‐c group than in the INF‐a/b group (hazard ratio 2.47). INF‐c was a significant risk factor for peritoneal recurrences in most subgroups including age, sex, macroscopic type, tumor differentiation, and disease stage, and whether the postoperative treatment was given. Multivariate analysis identified INF‐c as an independent risk factor for peritoneal recurrences. The cumulative liver recurrence rate was significantly higher in the INF‐a/b group than in the INF‐c group (hazard ratio 3.44).

**Conclusions:**

INF may represent an important predictor of recurrence patterns after curative resection of stage II/III gastric cancer.

## 1 INTRODUCTION

Gastric cancer is a common malignant tumor that is the second cause of all cancer deaths worldwide.^1^ Patients with stage I gastric cancer who undergo endoscopic or surgical resection can expect an excellent prognosis.^2^ On the contrary, individuals with stage II/III gastric cancer suffer more frequently from recurrences even if they undergo radical resection and adjuvant treatment.^3^, ^4^ To detect recurrences early and commence treatment, it is important to predict the sites of recurrences. If physicians predict recurrence sites accurately, the schedule and methods of postoperative surveillance can be optimized.

We recently reported that the pathological infiltrative pattern (INF) was closely related to sites of initial recurrence after curative resection of gastric cancer.^5^ In that report, patients with the infiltrative growth type had a significantly high risk of peritoneal recurrences, whereas those with the noninfiltrative type had a significantly high risk of hepatic recurrences.^5^ However, the study suffered from several limitations including being a single institution study with a small sample size, using patient data obtained over a prolonged period, and clinicopathologic differences between the patient groups that were compared.

To overcome these problems, we analyzed data from a multicenter integrated database of patients operated during the 5 years between 2010 and 2014 and made comparisons after propensity score matching. The aim of this study was to verify our findings regarding the relationship between the INF and recurrence patterns in patients with stage II/III gastric cancer.

## 2 PATIENTS AND METHODS

### 2.1 Patients

**Figure 1:**
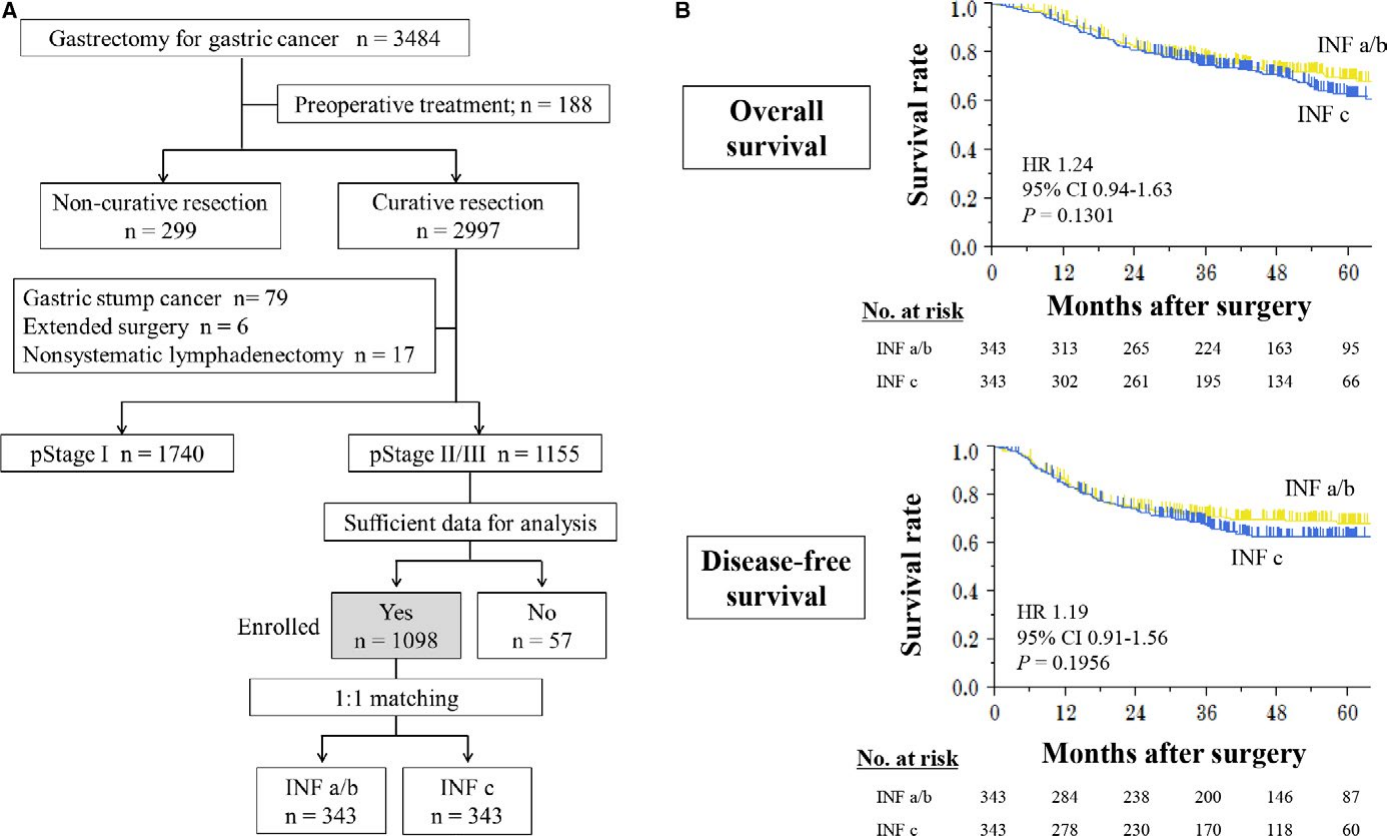
A, Flowchart of patient enrollment. B, Overall and disease‐free survival curves according to INF groups after adjustment using propensity score matching

Clinical data of 3484 patients who underwent gastrectomy for gastric cancer between January 2010 and December 2014 were retrospectively collected from medical records at nine institutions. Of these, we selected 1098 patients for analysis according to the following inclusion criteria: no preoperative treatment, R0 gastrectomy with systematic lymphadenectomy performed according to the Japanese Gastric Cancer Treatment Guidelines,^6^ pathologically diagnosed as stage II or III gastric cancer according to the TNM Classification of Malignant Tumors, 8th Edition,^7^ and sufficient data for analysis (Figure 1A). Patients with gastric stump cancer and those who underwent extended surgery (eg, pancreaticoduodenectomy and Appleby's procedure) were excluded. This study conformed to the ethical guidelines of the World Medical Association Declaration of Helsinki—Ethical Principles for Medical Research Involving Human Subjects. Patients provided written informed consent for surgery and use of clinical data as required by the Institutional Review Board at each participating institute.

### 2.2 Definition of pathological INF

Pathological diagnosis was determined by two institutional pathologists using paraffin sections stained with hematoxylin and eosin. The pathological INF types were classified into either INF‐a (expansive growth having a distinct border with the surrounding tissues), INF‐b (intermediate type), or INF‐c (infiltrative growth having no distinct border with the surrounding tissues), according to the Japanese Classification of Gastric Carcinoma as shown in Figure S1 and in previous reports.^5^, ^8^

### 2.3 Surgery and postoperative management

Patients underwent gastrectomy with systematic lymphadenectomy according to the Japanese Gastric Cancer Treatment Guidelines,^6^ and the reconstruction method was selected at the surgeon's discretion. Patients received postoperative follow‐up for 5 years or until recurrence that included physical examinations and laboratory tests including serum tumor markers every 3 months, and enhanced computed tomography (chest and abdominal cavity) once every 6 months, and upper gastrointestinal endoscopy at 1, 3, and 5 postoperative years as described in the Japanese Gastric Cancer Treatment Guidelines 6. Disease recurrences were diagnosed based on radiological or pathological findings, with serum tumor markers playing an adjunctive role.^6^ Twelve months of S‐1 (an oral fluoropyrimidine derivative) monotherapy or 6 months of capecitabine plus oxaliplatin has been recommended to all patients as postoperative adjuvant treatment unless contraindicated by a patient's condition or patient refusal.^9^, ^10^, ^11^ Treatment after recurrences was determined according to the evidence available at the time of treatment, according to the patient's condition, and with the patient's consent.

### 2.4 Propensity score matching

We employed propensity score matching to balance more strictly essential variables for the comparison analyses between INF‐c (invasive growth type) and INF‐a/b (noninvasive type). Propensity scores were estimated using a logistic regression model based on age, sex, tumor location, type of gastrectomy, disease stage, and adjuvant chemotherapy. Age and sex were included in the variables for the matching as the most fundamental demographics. Since tumor location affects biological properties of gastric cancer and extent of resection, it also was included. Type of gastrectomy should be balanced because it can influence on postoperative nutritional status and chemotherapy tolerability. Lastly, disease stage and adjuvant chemotherapy were major relevant factors to the main point of analysis in the present study (postoperative prognosis and recurrences), and thus, they were used for the matching. One‐to‐one matching without replacement was performed using a 0.1 caliper width, and the resulting score‐matched pairs were used in subsequent analyses.

### 2.5 Statistical analysis

The chi‐square and Mann‐Whitney tests were used to compare the two groups. Overall and disease‐free survival rates were calculated using the Kaplan‐Meier method, and the difference between survival curves was evaluated using the log‐rank test. Risk factors for peritoneal recurrences were evaluated using binomial logistic regression analysis. *P* < 0.05 was considered statistically significant. All statistical analyses were performed using JMP 13 software (SAS Institute Inc, Cary, NC, USA).

## 3 RESULTS

### 3.1 INF and clinical signatures before matching

Before propensity score matching, 707 and 391 patients were classified in the INF‐a/b and INF‐c groups, respectively. As shown in Table 1, there were significant differences between the INF‐a/b and INF‐c groups in age, sex distribution, tumor location, type of gastrectomy, pathological stage, and administration rate of postoperative adjuvant chemotherapy. Overall, survival time was significantly shorter in the INF‐c group than in the INF‐a/b group (hazard ratio [HR] 1.91, 95% confidence interval [CI] 1.46‐2.50, *P* < 0.0001; Figure S2A). Patients in the INF‐c group were more likely to have shorter disease‐free survival times compared with those in the INF‐a/b group (HR 1.64, 95% CI 1.32‐2.05, *P* < 0.0001; Figure S2B). In the multivariate analysis, INF‐c was identified as an independent risk factor for peritoneal recurrence (odds ratio, 1.98; 95% CI 1.33‐2.99; *P* = 0.0007) along with Borrmann type 4/5 tumor, pT4, lymphatic involvement, lymph node metastasis, and stage III (Table S1).

**Table 1:** Patient characteristics before and after propensity score matching

Characteristics	Unmatched comparison			Matched comparison		
	INF‐a/b (n = 707)	INF‐c (n = 391)	*P*	INF‐a/b (n = 343)	INF‐c (n = 343)	*P*
Age (years), mean ± SD	70.1 ± 9.6	66.7 ± 11.6	<0.0001	67.9 ± 9.86	67.9 ± 10.5	0.9761
Sex (male/female)	524/183	243/148	<0.0001	228/115	230/113	0.9354
Tumor location						
Entire	14 (2.0)	29 (7.4)	<0.0001	11 (3.21)	12 (3.50)	0.9956
Upper third	193 (27.3)	88 (22.5)		85 (24.8)	86 (25.1)	
Middle third	240 (34.0)	144 (36.8)		128 (37.3)	128 (37.3)	
Lower third	260 (36.8)	130 (33.3)		119 (34.7)	117 (34.1)	
Macroscopic type						
Type 4/5	21 (3.0)	64 (16.4)	<0.0001	10 (2.9)	44 (12.8)	<0.0001
Others	686 (97.0)	327 (83.6)		333 (97.1)	299 (87.2)	
Type of gastrectomy						
Total gastrectomy	250 (35.4)	172 (44.0)	0.0053	140 (40.8)	141 (41.1)	1.0000
Partial gastrectomy	457 (64.6)	219 (56.0)		203 (59.2)	202 (58.9)	
Surgical procedure						
Open	652 (92.2)	351 (89.8)	0.1791	320 (93.3)	305 (88.9)	0.0596
Laparoscopic	55 (7.8)	40 (10.2)		23 (6.7)	38 (11.1)	
Extent of lymph node dissection						
Non‐D2	148 (20.9)	89 (22.8)	0.4819	50 (14.6)	82 (23.9)	0.0019
D2	559 (79.1)	302 (77.2)		293 (85.4)	261 (76.1)	
Dissected lymph nodes, mean ±SD	37.7 ± 16.6	37.7 ± 15.4	0.9895	39.6 ± 15.8	37.4 ± 15.3	0.0639
Postoperative complication^a^ (%)	29.7	24.0	0.0486	29.2	24.2	0.1671
T factor						
pT1	42 (5.94)	7 (1.79)	<0.0001	11 (3.21)	7 (2.04)	<0.0001
pT2	123 (17.4)	25 (6.39)		50 (14.6)	25 (7.29)	
pT3	310 (43.9)	100 (25.6)		143 (41.7)	94 (27.4)	
pT4	232 (32.8)	259 (66.2)		139 (40.5)	217 (63.3)	
N factor						
pN0	137 (19.4)	94 (24.0)	<0.0001	48 (14.0)	88 (25.7)	0.0009
pN1	207 (29.3)	73 (18.7)		77 (22.5)	66 (19.2)	
pN2	211 (29.8)	91 (23.3)		110 (32.1)	83 (24.2)	
pN3	152 (21.5)	133 (34.0)		108 (31.5)	106 (30.9)	
Lymphatic invasion						
Absent	67 (9.5)	46 (11.8)	0.2541	27 (7.9)	40 (11.7)	0.1222
Present	640 (90.5)	345 (88.2)		316 (92.1)	303 (88.3)	
Venous invasion						
Absent	199 (28.1)	135 (34.5)	0.0286	79 (23.0)	119 (34.7)	0.0010
Present	508 (71.9)	256 (65.5)		264 (77.0)	224 (65.3)	
Pathological stage						
IIA	201 (28.4)	57 (14.6)	<0.0001	56 (16.3)	57 (16.6)	0.9430
IIB	161 (22.8)	75 (19.2)		64 (18.7)	69 (20.1)	
IIIA	200 (28.3)	126 (32.2)		122 (35.6)	112 (32.7)	
IIIB	105 (14.9)	89 (22.8)		69 (20.1)	70 (20.4)	
IIIC	40 (5.66)	44 (11.3)		32 (9.33)	35 (10.2)	
Adjuvant chemotherapy						
Absent	291 (41.1)	124 (31.7)	0.0022	109 (31.8)	116 (33.8)	0.6256
Present	416 (58.8)	267 (68.3)		234 (68.2)	227 (66.2)	

SD, standard deviation.

a Postoperative complications included those of Clavien‐Dindo classifications II‐V.

### 3.2 Patient characteristics after matching

After propensity score matching, 686 patients (343 for each) were classified in the INF‐a/b and INF‐c groups, and age, sex distribution, tumor location, type of gastrectomy, pathological stage, and administration rate of postoperative adjuvant chemotherapy were well balanced (Table 1). There were 461 patients who underwent postoperative adjuvant chemotherapy, and 415 (90%) patients received S‐1 monotherapy. The INF‐c group had a significantly greater proportion of Borrmann 4/5 type tumors, undifferentiated tumors, and advanced pT, whereas the INF‐a/b group had elevated preoperative serum CEA levels, vascular invasion, and pathological lymph node metastasis (Table S2).

### 3.3 Prognostic impact of INF in stage II/III gastric cancer after matching

After propensity score matching, survival differences between the INF‐a/b and INF‐c groups were reduced. There were no significant differences in overall survival and disease‐free survival between the INF‐a/b and INF‐c groups (Figure 1B).

### 3.4 Association between INF and recurrence patterns

**Figure 2:**
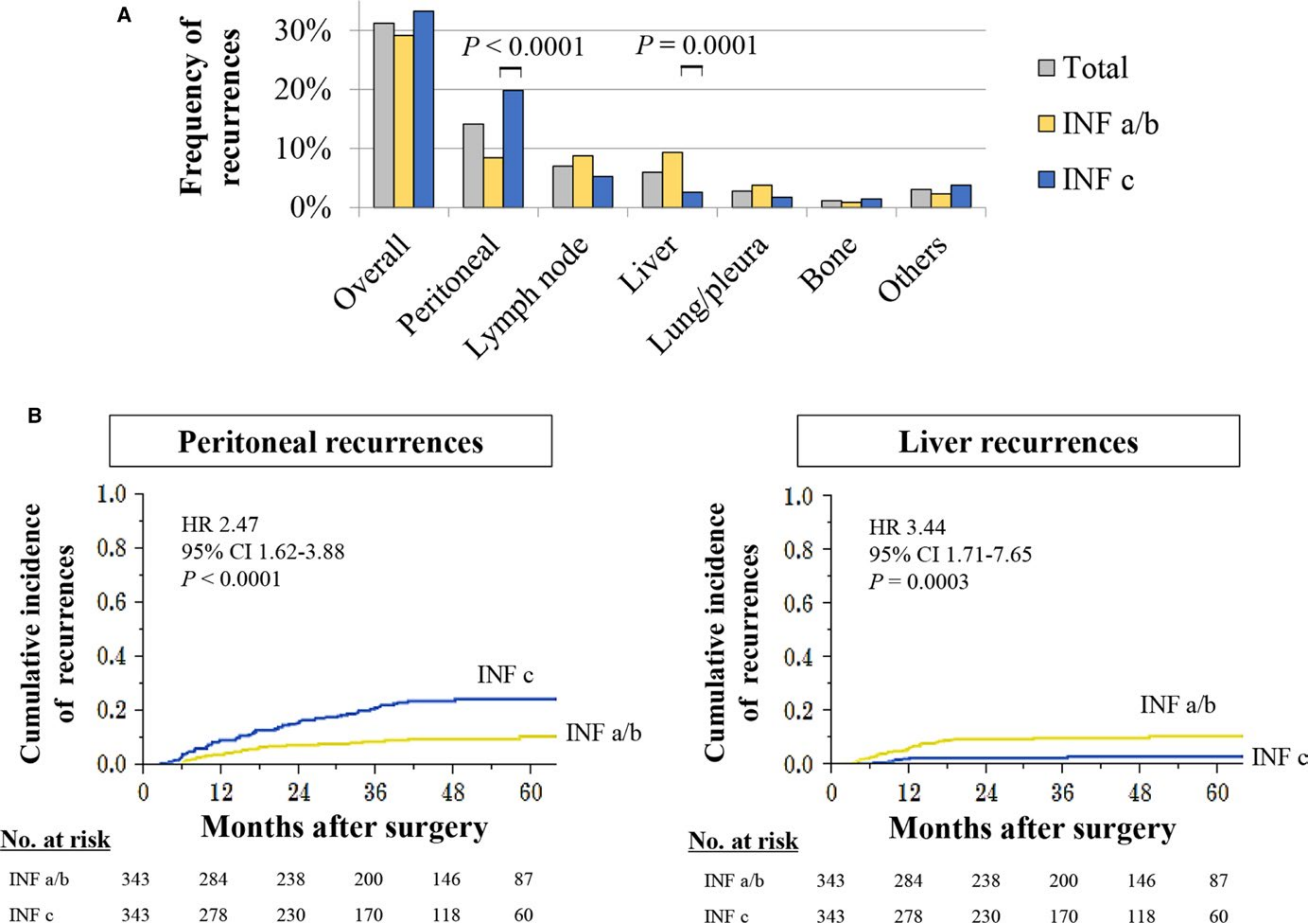
Recurrence patterns and INF. A, Frequencies of the sites of initial recurrence in the INF‐a/b and the INF‐c groups. B, The cumulative incidence of peritoneal recurrence according to each INF group. C, The cumulative incidence of liver recurrence according to each INF group

The frequency of initial recurrence sites is depicted in Figure 2A. The overall recurrence rates of the INF‐a/b and INF‐c groups were equivalent. In the INF‐a/b group, the frequency of recurrences at the peritoneum, lymph node, and liver was similar and each accounted for approximately 30% of the total recurrences. In the INF‐c group, the peritoneum was the most frequent site and it accounted for 60% of the total recurrences. The prevalence of peritoneal recurrences was significantly greater in the INF‐c group than in the INF‐a/b group. On the contrary, liver recurrences were more commonly observed in the INF‐a/b group.

The cumulative peritoneal recurrence rate was significantly higher in the INF‐c group than in the INF‐a/b group (HR 2.47, 95% CI 1.62‐3.88, *P* < 0.0001; Figure 2B). In univariate analysis, macroscopic tumor size ≥50 mm, Borrmann type 4/5 tumor, pT4, undifferentiated tumor, lymphatic involvement, vascular invasion, INF‐c, and stage III were identified as significant risk factors for peritoneal recurrence. Multivariate analysis identified INF‐c as an independent risk factor for peritoneal recurrence after curative gastrectomy (odds ratio, 1.70; 95% CI 1.06‐2.78; *P* = 0.0270) along with Borrmann type 4/5 tumor, pT4, and stage III (Table 2). In contrast, the cumulative liver recurrence rate was significantly higher in the INF‐a/b group than in the INF‐c group (HR 3.44, 95% CI 1.71‐7.65, *P = *0.0003; Figure 2C).

**Table 2:** Predictive factors of peritoneal recurrence in 686 patients with stage II/III gastric cancer

Variables	P‐rec (‐)	P‐rec (+)	Univariate		Multivariable	
			*P* value	OR	95% CI	*P* value
Age						
<65 years	202	29	0.1220			
≥65 years	387	68				
Sex						
Male	397	61	0.5512			
Female	192	36				
CEA						
≤5 ng/mL	455	77	0.7899			
>5 ng/mL	108	17				
CA19‐9						
≤37 IU/mL	470	75	0.0906			
>37 IU/mL	88	19				
Tumor location						
Lower third	205	31	0.5374			
Others	384	66				
Tumor size						
<50 mm	281	27	<0.0001	1.23	0.77‐2.02	0.3838
≥50 mm	307	70				
Macroscopic type						
Others	556	76	<0.0001	3.01	1.76‐4.97	<0.0001
Borrmann 4/5	33	21				
Multifocal lesions						
Absent	567	94	0.6454			
Present	22	3				
Tumor depth						
pT1‐3	316	14	<0.0001	3.68	2.09‐6.97	<0.0001
pT4	273	83				
Differentiation						
Differentiated	243	24	0.0013	1.44	0.89‐2.42	0.1404
Undifferentiated	346	73				
Lymphatic involvement						
Absent	63	4	0.0168	1.73	0.68‐5.88	0.2738
Present	526	93				
Vascular invasion						
Absent	177	21	0.0356	1.27	0.77‐2.17	0.3543
Present	412	76				
Infiltrative growth						
INF‐a/b	314	29	<0.0001	1.70	1.06‐2.78	0.0270
INF‐c	275	68				
Lymph node metastasis						
Absent	121	15	0.1420			
Present	468	82				
UICC stage						
II	235	11	<0.0001	2.86	1.48‐6.00	0.0012
III	354	86				
Adjuvant chemotherapy						
Absent	198	27	0.8096			
Present	391	70				

CA19‐9, carbohydrate antigen 19‐9; CEA, carcinoembryonic antigen; CI, confidence interval; INF, tumor infiltrative pattern; OR, odds ratio; UICC, Union for International Cancer Control.

### 3.5 Further evaluation of INF‐c as a risk factor of peritoneal recurrences

**Figure 3:**
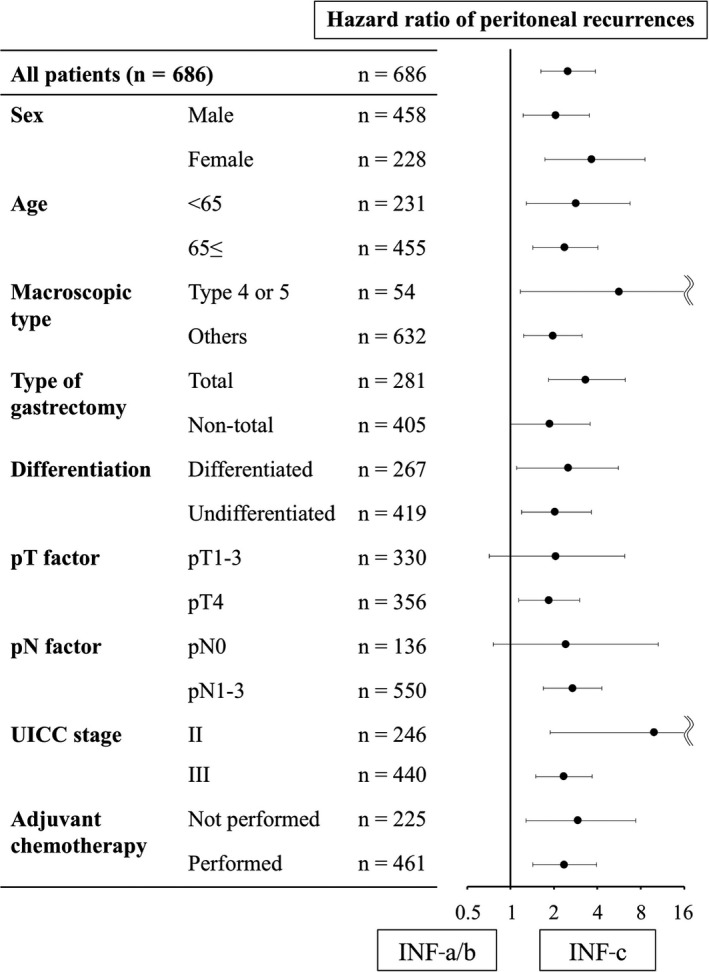
A forest plot evaluating the impact of INF‐c on peritoneal recurrences

A forest plot to evaluate the impact of INF‐c on peritoneal recurrences is shown in Figure 3. INF‐c was a significant risk factor for peritoneal recurrences in most subgroups including age, sex, macroscopic type, tumor differentiation, and disease stage. Of note, INF‐c had a significant influence on peritoneal recurrences both in patients who underwent surgery alone (n = 225, HR 2.90, 95% CI 1.28‐7.38, *P* = 0.0010) and in those who underwent postoperative adjuvant chemotherapy (n = 461, HR 2.34, 95% CI 1.43‐3.95, *P* = 0.0006).

## 4 DISCUSSION

In East Asia, pathological INF has long been routinely evaluated in surgically resected specimens.^12^, ^13^ INF can be easily determined using only hematoxylin and eosin‐stained sections.^12^, ^14^ However, there have been a few recent studies focusing on the clinical significance of INF in gastric cancer.^13^, ^14^, ^15^ In the present study, we revalidated the impact of pathological INF on prognosis and recurrence patterns after curative gastrectomy in patients with stage II/III gastric cancer using a large multicenter dataset. Using propensity score matching, it was revealed that there was no difference in overall survival time, disease‐free survival time, and overall recurrence rates between the INF‐a/b and INF‐c groups. In contrast, significant differences in recurrence patterns were detected between the INF‐a/b and INF‐c groups, and INF‐c was found to be an independent risk factor for peritoneal recurrences.

Our findings suggest that INF‐c type gastric cancer tends to grow out of the stomach wall directly by skipping the lymphatic and blood vascular systems, and that INF‐a/b type gastric cancer causes lymphatic involvement and vascular invasion in parallel with growth outside the stomach wall. It has been reported that INF‐c is closely linked to other risk factors for peritoneal metastasis such as Borrmann type 4 tumors, poorly differentiated tumors, and serosal invasion.^13^, ^14^, ^15^, ^16^ However, our multivariate analysis revealed that INF‐c was an independent risk factor for peritoneal recurrences. Since there is a certain correlation between the macroscopic type and pathological INF, clinicians believe that INF‐c groups are mostly type 4/5 tumors and associate with peritoneal recurrences. However, physicians sometimes experience cases of INF‐c gastric cancer with non‐linitis plastica type macroscopic appearance and/or well‐differentiated type histology.^2^ In fact, we showed here that 83.6% (unmatched) and 87.2% (matched) of INF‐c tumors had type 4/5 macroscopic appearance. Moreover, INF‐c was an independent risk factor for peritoneal recurrences in multivariable analysis considering a confounding between macroscopic tumor type and INF. These findings may make the divergence between clinical belief and the actual distribution of INF in each macroscopic tumor type. Thus, INF is a clinically useful predictor of recurrence patterns after gastrectomy.

For patients at high risk of postoperative recurrence, adjuvant chemotherapy is recognized as the standard of care in the Far East.^17^, ^18^ In our patient cohort, S‐1 monotherapy comprised the majority (90%) of treatment regimens for adjuvant therapy. It has been suggested that S‐1 adjuvant chemotherapy mainly suppresses peritoneal recurrences based on results of the Adjuvant Chemotherapy Trial of TS‐1 for Gastric Cancer (ACTS‐GC) phase III clinical trial.^19^ However, results of a subgroup analysis showed that INF‐c was a significant risk factor for peritoneal recurrences irrespective of whether the adjuvant chemotherapy was given, and highlighted the utility of INF‐c as an indicator to screen peritoneal recurrences even in patients who underwent S‐1 adjuvant treatment. Development of postoperative treatments that excel in controlling peritoneal recurrences shed a new light on patients with INF‐c gastric cancer.

Accurate prediction of recurrence sites is extremely important in postoperative follow‐up because early detection of recurrences will be possible by conducting appropriate surveillance.^20^, ^21^ By detecting recurrent lesions early, the first‐line treatment for recurrences can be initiated early. In the current Japanese Treatment Guideline of Gastric Cancer, follow‐up methods after curative resection are recommended uniformly according to only pathological disease stage.^6^ In the case of INF‐c, patients who are at high risk for a peritoneal recurrence but at low risk for a hematogenous metastasis, a regular abdominal computed tomography (CT), ultrasound, or digital rectal examination is advisable to detect malignant ascites fluids and peritoneal nodules. For patients with suspected peritoneal recurrences, staging laparoscopy or ascites puncture cytology is considered accordingly. Meanwhile, for patients with INF‐a/b type gastric cancer, who are at higher risk of hematogenous and nodal metastasis, broad range (cervical to abdominal) contrast CT scan or positron‐emission tomography might be given preference to detect recurrences early. Once evidence for selecting appropriate anticancer drugs or treatments based on the pattern of metastasis can be established, INF can be a candidate selection factor of treatment methods.

This study also has some limitations. It was a retrospective study. It was difficult to completely eliminate the pathologists’ subjectivity in the evaluation of INF. Furthermore, lack of information on postrecurrence treatment restricted the discussion. Nevertheless, our preceding thesis was successfully reproduced using a large multicenter database, indicating that the value of INF in predicting recurrence patterns was enhanced.

In conclusion, pathological INF represents an important predictive factor for recurrence patterns after radical resection of stage II/III gastric cancer and may guide clinicians in providing appropriate postoperative management.

## RESEARCH INVOLVING HUMAN PARTICIPANTS INFORMED CONSENT

This study conforms to the ethical guidelines of the World Medical Association Declaration of Helsinki—Ethical Principles for Medical Research Involving Human Subjects, and written informed consent for surgery and the use of clinical data were obtained from all patients as required by the Institutional Review Board of all participating institutes.

## CONFLICTS OF INTEREST

None declared.

## References

[1] Siegel RL , Miller KD , Jemal A . Cancer statistics, 2017. CA Cancer J Clin. 2017;67:7‐30. 28055103 10.3322/caac.21387

[2] Van Cutsem E , Sagaert X , Topal B , Haustermans K , Prenen H . Gastric cancer. Lancet. 2016;388:2654‐2664. 27156933 10.1016/S0140-6736(16)30354-3

[3] Kanda M , Tanaka C , Kobayashi D , et al. Proposal of the coagulation score as a predictor for short‐term and long‐term outcomes of patients with resectable gastric cancer. Ann Surg Oncol. 2017;24:502‐509. 27600621 10.1245/s10434-016-5544-1

[4] Shen L , Shan YS , Hu HM , et al. Management of gastric cancer in Asia: resource‐stratified guidelines. Lancet Oncol. 2013;14:e535‐e547. 24176572 10.1016/S1470-2045(13)70436-4

[5] Kanda M , Mizuno A , Fujii T , et al. Tumor infiltrative pattern predicts sites of recurrence after curative gastrectomy for stages 2 and 3 gastric cancer. Ann Surg Oncol. 2016;23:1934‐1940. 26847679 10.1245/s10434-016-5102-x

[6] Japanese gastric cancer treatment guidelines 2014 (ver. 4). Gastric Cancer. 2017;20:1‐19. 10.1007/s10120-016-0622-4 PMC5215069 27342689

[7] Liu JY , Peng CW , Yang XJ , Huang CQ , Li Y . The prognosis role of AJCC/UICC 8(th) edition staging system in gastric cancer, a retrospective analysis. Am J Transl Res. 2018;10:292‐303. 29423014 PMC5801367

[8] Japanese classification of gastric carcinoma: 3rd English edition. Gastric Cancer. 2011;14:101‐112. 21573743 10.1007/s10120-011-0041-5

[9] Kanda M , Murotani K , Kobayashi D , et al. Postoperative adjuvant chemotherapy with S‐1 alters recurrence patterns and prognostic factors among patients with stage II/III gastric cancer: A propensity score matching analysis. Surgery. 2015;158:1573‐1580. 26120068 10.1016/j.surg.2015.05.017

[10] Noh SH , Park SR , Yang HK , et al. Adjuvant capecitabine plus oxaliplatin for gastric cancer after D2 gastrectomy (CLASSIC): 5‐year follow‐up of an open‐label, randomised phase 3 trial. Lancet Oncol. 2014;15:1389‐1396. 25439693 10.1016/S1470-2045(14)70473-5

[11] Kanda M , Kodera Y , Sakamoto J . Updated evidence on adjuvant treatments for gastric cancer. Expert Rev Gastroenterol Hepatol. 2015;9(12):1549‐1560. 26414453 10.1586/17474124.2015.1094373

[12] Maehara Y , Oshiro T , Adachi Y , Ohno S , Akazawa K , Sugimachi K . Growth pattern and prognosis of gastric cancer invading the subserosa. J Surg Oncol. 1994;55:203‐208. 8159002 10.1002/jso.2930550402

[13] Saito H , Miyatani K , Takaya S , et al. Tumor infiltration pattern into the surrounding tissue has prognostic significance in advanced gastric cancer. Virchows Arch.. 2015;467(5):519‐523. 26277483 10.1007/s00428-015-1811-y

[14] Song KY , Hur H , Jung CK , et al. Impact of tumor infiltration pattern into the surrounding tissue on prognosis of the subserosal gastric cancer (pT2b). Eur J Surg Oncol. 2010;36:563‐567. 20462730 10.1016/j.ejso.2010.04.006

[15] Huang B , Sun Z , Wang Z , et al. Factors associated with peritoneal metastasis in non‐serosa‐invasive gastric cancer: a retrospective study of a prospectively‐collected database. BMC Cancer. 2013;13:57. 23379700 10.1186/1471-2407-13-57 PMC3641004

[16] Nishida T , Tanaka S , Haruma K , Yoshihara M , Sumii K , Kajiyama G . Histologic grade and cellular proliferation at the deepest invasive portion correlate with the high malignancy of submucosal invasive gastric carcinoma. Oncology. 1995;52:340‐346. 7777250 10.1159/000227486

[17] Jacome AA , Sankarankutty AK , dos Santos JS . Adjuvant therapy for gastric cancer: what have we learned since INT0116? World J Gastroenterol. 2015;21:3850‐3859. 25852269 10.3748/wjg.v21.i13.3850 PMC4385531

[18] Wong RK , Jang R , Darling G . Postoperative chemoradiotherapy vs. preoperative chemoradiotherapy for locally advanced (operable) gastric cancer: clarifying the role and technique of radiotherapy. J Gastrointest Oncol. 2015;6:89‐107. 25642342 10.3978/j.issn.2078-6891.2014.089 PMC4294828

[19] Sasako M , Sakuramoto S , Katai H , et al. Five‐year outcomes of a randomized phase III trial comparing adjuvant chemotherapy with S‐1 versus surgery alone in stage II or III gastric cancer. J Clin Oncol. 2011;29:4387‐4393. 22010012 10.1200/JCO.2011.36.5908

[20] Liu X , Zhang X , Zhang Z , et al. Plasma microRNA‐based signatures to predict 3‐year postoperative recurrence risk for stage II and III gastric cancer. Int J Cancer. 2017;141:2093‐2102. 28722210 10.1002/ijc.30895

[21] Kanda M , Tanaka H , Shimizu D , et al. SYT7 acts as a driver of hepatic metastasis formation of gastric cancer cells. Oncogene. 2018;37(39):5355‐5366. 29858600 10.1038/s41388-018-0335-8

