# Supplementary figures and images for: Pathological tumor infiltrative pattern and sites of initial recurrence in stage II/III gastric cancer: Propensity score matching analysis of a multi‐institutional dataset
Source: Cancer Med. 2018 Nov 8;7(12):6020–9. doi: 10.1002/cam4.1868 (PMC6308072; doi:10.1002/cam4.1868)

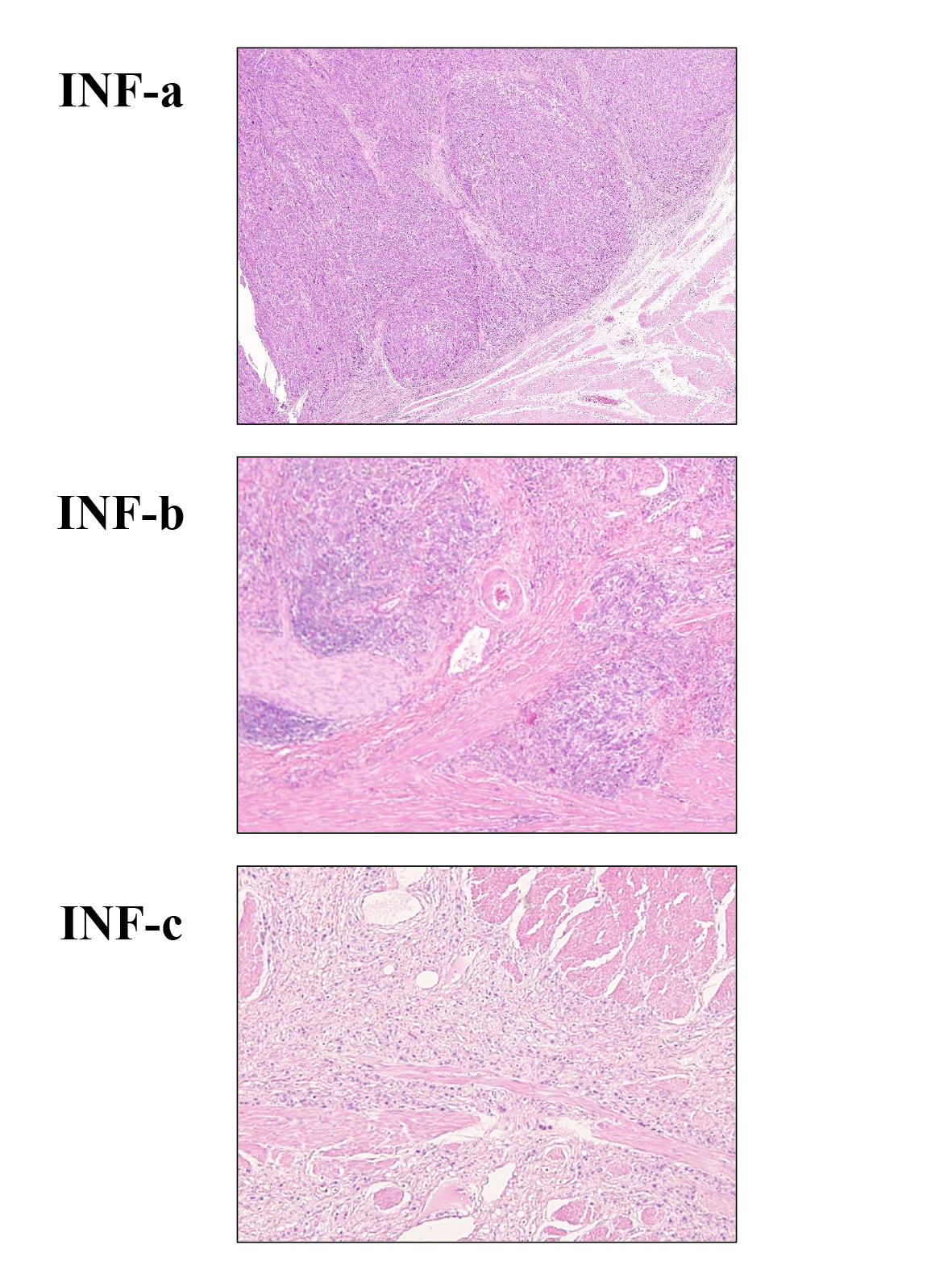

Supplement: Supplementary file 1 [file CAM4-7-6020-s001.tif]

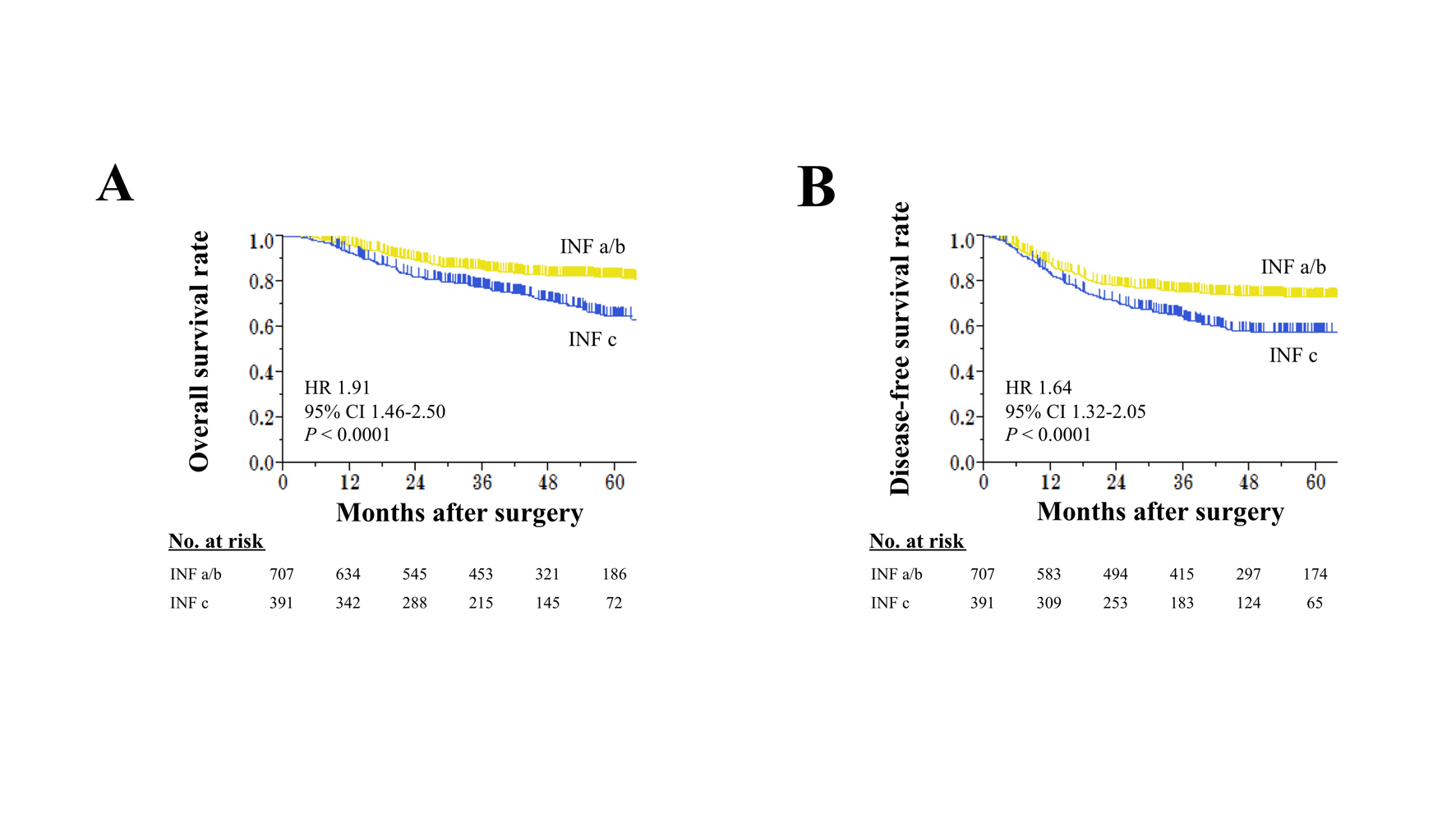

Supplement: Supplementary file 2 [file CAM4-7-6020-s002.tif]
