# Supplementary material for: Pathological tumor infiltrative pattern and sites of initial recurrence in stage II/III gastric cancer: Propensity score matching analysis of a multi‐institutional dataset
Source: Cancer Med. 2018 Nov 8;7(12):6020–9. doi: 10.1002/cam4.1868 (PMC6308072; doi:10.1002/cam4.1868)
Supplement: Supplementary file 3 [file CAM4-7-6020-s003.docx]

**Supplementary Table 1.** Predictive factors of peritoneal recurrence in 1098 patients with stage II/III gastric cancer.

| **Variables** | | **P-rec (-)** | **P-rec (+)** | **Univariate** | **Multivariable** | | |
| --- | --- | --- | --- | --- | --- | --- | --- |
|  |  |  |  | ***P* value** | **OR** | **95%CI** | ***P* value** |
| Age | < 65 years  ≥ 65 years | 288  673 | 44  93 | 0.7997 |  |  |  |
| Sex | Male  Female | 685  276 | 82  55 | 0.0225 | 0.88 | 0.62 – 1.27 | 0.5089 |
| CEA | ≤ 5 ng/ml  > 5 ng/ml | 720  183 | 109  23 | 0.8745 |  |  |  |
| CA19-9 | ≤ 37 IU/ml  > 37 IU/ml | 747  147 | 108  24 | 0.1917 |  |  |  |
| Tumor location | Lower third  Others | 344  617 | 46  91 | 0.5489 |  |  |  |
| Tumor size | < 50 mm  ≥ 50 mm | 475  486 | 43  94 | <0.0001 | 1.20 | 0.82 – 1.78 | 0.3536 |
| Macroscopic type | Others  Borrmann 4/5 | 908  53 | 105  32 | <0.0001 | 2.68 | 1.71 - 4.12 | <0.0001 |
| Multifocal lesions | Absent  Present | 912  48 | 132  5 | 0.4297 |  |  |  |
| Tumor depth | pT1-3  pT4 | 579  382 | 28  109 | <0.0001 | 2.20 | 1.41 – 3.56 | 0.0004 |
| Differentiation | Differentiated Undifferentiated | 451  510 | 36  101 | <0.0001 | 1.26 | 0.83 – 1.95 | 0.2827 |
| Lymphatic involvement | Absent  Present | 107  854 | 6  131 | 0.0046 | 2.33 | 1.08 – 6.08 | 0.0291 |
| Venous invasion | Absent  Present | 298  663 | 36  101 | 0.1113 |  |  |  |
| Infiltrative growth | INF a/b  INF c | 660  301 | 47  90 | <0.0001 | 1.98 | 1.33 - 2.99 | 0.0007 |
| Lymph node metastasis | Absent  Present | 210  751 | 21  116 | 0.0307 | 0.36 | 0.17 – 0.82 | 0.0154 |
| UICC stage | II  III | 472  489 | 22  115 | <0.0001 | 5.44 | 2.49 – 12.1 | <0.0001 |
| Adjuvant chemotherapy | Absent  Present | 374  587 | 41  96 | 0.3316 |  |  |  |

*OR*, odds ratio; *CI*, confidence interval; *CEA*, carcinoembryonic antigen; *CA19-9*, carbohydrate antigen 19-9; *INF*, tumor infiltrative pattern; *UICC*, Union for International Cancer Control.
