# Supplementary material for: Pathological tumor infiltrative pattern and sites of initial recurrence in stage II/III gastric cancer: Propensity score matching analysis of a multi‐institutional dataset
Source: Cancer Med. 2018 Nov 8;7(12):6020–9. doi: 10.1002/cam4.1868 (PMC6308072; doi:10.1002/cam4.1868)
Supplement: Supplementary file 4 [file CAM4-7-6020-s004.docx]

**Supplemental Table 2.** Association between infiltrative type growth and clinicopathological characteristics in 686 patients with stage II/III gastric cancer.

| **Variables** | **INF a/b** | **INF c** | ***P* value** |
| --- | --- | --- | --- |
| Age  < 65 years  ≥ 65 years | 117  226 | 114  229 | 0.8717 |
| Sex  Male  Female | 228  115 | 230  113 | 0.9354 |
| CEA (ng/ml)  ≤ 5  > 5 | 251  75 | 281  50 | 0.0127 |
| CA19-9 (IU/ml)  ≤ 37  > 37 | 265  58 | 280  49 | 0.3413 |
| Tumor location  Entire  Upper third  Middle third  Lower third | 11  85  128  119 | 12  86  128  117 | 0.9956 |
| Tumor size (mm)  < 50  ≥ 50 | 148  194 | 160  183 | 0.3985 |
| Macroscopic type  Others  Borrmann type 4/5 | 333  10 | 299  44 | <.0001 |
| Multifocal lesions  Absent  Present | 326  17 | 335  8 | 0.1013 |
| Tumor depth (UICC)  pT1  pT2  pT3  pT4 | 11  50  143  139 | 7  25  94  217 | <.0001 |
| Differentiation  Differentiated  Undifferentiated | 196  147 | 71  272 | <0.001 |
| Lymphatic involvement  Absent  Present | 27  316 | 40  303 | 0.1222 |
| Vessel invasion  Absent  Present | 79  264 | 119  224 | 0.0010 |
| Lymph node metastasis  Absent  Present | 48  295 | 88  255 | 0.0002 |
| UICC stage  II  III | 120  223 | 126  217 | 0.6906 |

*INF*, tumor infiltrative pattern; *CEA,* carcinoembryonic antigen; *CA19-9*, carbohydrate antigen 19-9; *UICC*, Union for International Cancer Control.
